# A bioprinted complex tissue model for myotendinous junction with biochemical and biophysical cues

**DOI:** 10.1002/btm2.10321

**Published:** 2022-04-05

**Authors:** Won Jin Kim, Geun Hyung Kim

**Affiliations:** ^1^ Department of Biomechatronic Engineering College of Biotechnology and Bioengineering, Sungkyunkwan University (SKKU) Suwon Republic of Korea; ^2^ Biomedical Institute for Convergence at SKKU (BICS), Sungkyunkwan University Suwon Republic of Korea

**Keywords:** 3D bioprinting, bioink, hASCs, myotendinous *junction*, tissue engineering

## Abstract

In the musculoskeletal system, the myotendinous junction (MTJ) is optimally designed from the aspect of force transmission generated from a muscle through a tendon onto the bone to induce movement. Although the MTJ is a key complex tissue in force transmission, the realistic fabrication, and formation of complex tissues can be limited. To obtain the MTJ construct, we prepared two bioinks, muscle‐ and tendon‐derived decellularized extracellular matrix (dECM), which can induce myogenic and tenogenic differentiation of human adipose‐derived stem cells (hASCs). By using a modified bioprinting process supplemented with a nozzle consisting of a single‐core channel and double‐sheath channels, we can achieve three different types of MTJ units, composed of muscle, tendon, and interface zones. Our results indicated that the bioprinted dECM‐based constructs induced hASCs to myogenic and tenogenic differentiation. In addition, a significantly higher MTJ‐associated gene expression was detected at the MTJ interface with a cell‐mixing zone than in the other interface models. Based on the results, the bioprinted MTJ model can be a potential platform for understanding the interaction between muscle and tendon cells, and even the bioprinting method can be extensively applied to obtain complex tissues.

## 1 INTRODUCTION

In the human body, most tissues have unique physical and biological functions, with tissues with physically differing properties being present to perform specific biological or biomechanical functions. Furthermore, heterogeneous tissues are connected by specific interfaces for performing complicated functions.^1^ The complex interfaces of orthopedic tissues can be divided into four types: cartilage–bone, ligament–bone, tendon–bone, and muscle–tendon interfaces.^2^, ^3^, ^4^ Because most orthopedic tissue interfaces are biomechanical connections between relatively soft and hard materials, with the application of a high external strain to the region, injury, or damage caused by the stress concentration generally occurs in the transition region.^5^ In particular, the myotendinous junction (MTJ) is important for transmitting force from a contracting muscle, through a tendon, to the bone, so that severe injuries, such as rotator cuff tears, Achilles tendon ruptures, and hamstring damage of the MTJ can occur as a result of repetitive and excessive loading or overtraining.^6^ Specific MTJ structural interfaces, which are formed by the interaction between tenocytes and myotubes, consist of three typical regions: (1) elastic muscle, (2) stiff tendon, and (3) a complicated cell matrix.^7^ The structure of the MTJ can be described as a tapered myofiber that can be entrenched in the tendon ECM.

Generally, the approach to heal an MTJ defect has involved the use of sutures, but this method has a high recurrence rate and induces the formation of fibrous scar tissue in the defective region.^8^, ^9^, ^10^ To overcome this problem, MTJ regeneration using tissue engineering strategies has been pursued as an alternative and, in particular, research using functional biomaterials with structurally and biochemically biomimetic properties in the tissues of muscles and tendons and their interfaces have been the subject of several studies.^11^, ^12^, ^13^, ^14^ Although significant progress has been made in MTJ regeneration through the use of biomaterials and printing technologies, engineered biomaterials showing the unique physical/biological characteristics of MTJ are still required to obtain a perfect junction structure. Furthermore, most of the research has been focused on biomaterials and processing techniques to regenerate muscle and tendon tissues, although the MTJ function is a critical component in force transmission through the muscle–tendon, much less research has been conducted on the regeneration of the muscle and tendon interface.^15^, ^16^

In general, to regenerate MTJ substitutes, a typical scaffold‐based strategy has been used. Using scaffold‐based regeneration with synthetic or acellularized biomaterials and biomimetic hydrogels with or without cells, several advantageous factors, such as controllable pore geometry and physical properties, can be adapted as engineering substitutes.^17^, ^18^ However, the rigid scaffold can cause a stress‐shielding effect on the cells for externally applied physical loading, eventually inducing low biological responses from the muscle and tendon cells.^5^, ^7^, ^19^ In addition, an acellular structure that could be used to regenerate tendon tissue still has limitations including poor cell infiltration owing to the highly dense fibrous matrix.^20^, ^21^, ^22^ In recent, electrospun nanofibers using polycaprolactone/chitosan/cellulose nanocrystal for regenerating tendon and ligament tissues were fabricated.^21^ The human tendon‐derived cells and human adipose‐derived stem cells (hASCs), seeded on the nanofibers, have been organized into anisotropic tendon tissues, highly expressing tenogenesis‐related genes. Although the successful induction to tenogenic differentiation was achieved, the cells infiltrated into the nanofibers have been observed after long culture period. Recently, to overcome scaffold limitations, a 3D bioprinting process has been used to obtain an MTJ construct that can mimic the physical structure and biofunctional characteristics of the native MTJ structure.^13^ To serve mechanically functional constructs, highly elastic polyurethane and C2C12‐containing hydrogel in the muscle tissue and stiff poly(ε‐caprolactone) and NIH/3 T3‐containing hydrogel in the tendon were applied. The hybrid construct demonstrated the characteristics of a mechanically heterogeneous MTJ scaffold, and the MTJ‐related genes at the interface were expressed well.

Herein, we suggest an innovative biofabrication process to obtain *an* in vitro MTJ unit (muscle‐MTJ‐tendon). To obtain the construct, two typical biomaterials, namely, (1) decellularized extracellular matrixes (dECMs) derived from porcine muscle and tendon, and (2) type I collagen were used. To attain the MTJ unit, we adapted a 3D bioprinting process supplemented with a newly designed core–sheath nozzle in which a single‐core channel and double‐sheath channels were composed. The high weight fraction of collagen served as a mechanical supporter in the core, while in the sheath, two hASC‐containing dECM‐based bioinks were used as biological cell‐differentiating niche components. Furthermore, to obtain aligned muscle and tendon tissue constructs, printing parameters, such as printing rates in the core and sheath and the collagen weight fraction in the core, were manipulated. In addition, we fabricated three different types of MTJ constructs to evaluate which physical interfacing shape of muscle and tendon cells can best mimic the native MTJ structure by measuring MTJ‐associated genes and mechanical properties.

## 2 EXPERIMENTAL SECTION

### 2.1 Preparation of cells and bioinks

To formulate cell‐containing bioinks, hASCs (Lonza, USA) were used. The cells were cultured in a culture medium (CM) consisting of Dulbecco's modified Eagle's medium‐low glucose (DMEM‐L; Sigma‐Aldrich), 10% fetal bovine serum (FBS; BioWest), and 1% penicillin–streptomycin (PS; Thermo‐Fisher Scientific) at 37°C under 5% CO_2_. The CM was changed every 2 days.

Before preparing the muscle‐specific and tendon‐specific bioinks, skeletal muscle, and tendon tissues, isolated from the lower limbs of Yorkshire pigs (female; 10–15 months old), were decellularized using previously described protocols, described in Supplementary Information.^23^, ^24^, ^25^

For the muscle bioink (M‐bioink), mdECM was dissolved in 0.1 M acetic acid solution and mixed with 10× enriched DMEM (Sigma‐Aldrich) at a ratio of 1:1. Fibronectin (2.5 mg/ml) and hASCs (2 × 10^7^ cells/ml) were mixed with the neutralized mdECM hydrogel (3 wt%) to obtain the M‐bioink.

For the tendon bioink (T‐bioink), tdECM was dissolved in 0.1 M acetic acid solution and mixed with 10× DMEM at a ratio of 1:1. T‐bioink was obtained by adding hASCs (2 × 10^7^ cells/mL) to the neutralized tdECM hydrogel (3 wt%).

For the collagen bioink, type I collagen sponge (MSbio, South Korea) was dissolved in 0.1 M acetic acid solution and mixed with 10× DMEM at a ratio of 1:1. The neutralized collagen hydrogel (3 wt%) was mixed with hASCs (2 × 10^7^ cells/ml).

For the core channel, type I collagen sponge was dissolved in 0.1 M acetic acid solution and mixed with 10× DMEM at a ratio of 1:1 to obtain collagen hydrogels of a range of concentrations (3, 4, 5, and 7 wt%).

To evaluate the myogenesis and tenogenesis of the stem cells, we performed two different cell cultures: (1) hASCs (5 × 10^3^ cells per cm^2^) were seeded onto tissue culture plates coated with cell‐free collagen, mdECM, and tdECM, and (2) three cell‐laden bioinks (collagen, mdECM, and tdECM) with the cell‐density (hASC, 2 × 10^7^ cells/ml) were cultured. The seeded cells and cell‐laden bioinks were cultured using the CM under 5% CO_2_ at 37°C. The CM was changed every 2 days.

### 2.2 Bioprinting conditions

To fabricate the muscle, tendon, and MTJ structures, the prepared core collagen hydrogel, collagen bioink, M‐bioink, and T‐bioink were printed using a 3D printing system (DTR3‐2210 T‐SG; DASA Robot) supplemented with a modified core/sheath nozzle with one core channel and two sheath channels, and a pneumatic pressure dispenser (AD‐3000C; Ugin‐tech). The printing conditions, including the printing barrel temperature (20°C), working plate temperature (35–38°C), and printing rate (5 mm/s), were fixed. The printed structures were rinsed three times with DPBS and then cultured in CM under 5% CO_2_ at 37°C. The CM was changed every 2 days.

### 2.3 Characterization of bioinks and cell‐containing structures

The rheological properties (storage modulus, G) of the cell‐containing bioinks were analyzed using a cone‐and‐plate geometry (diameter 40 mm, cone angle 4°, gap 150 μm) supplemented with a Bohlin Gemini HR Nano rotational rheometer (Malvern Instruments) conducted with a temperature sweep (strain 1%, frequency 1 Hz, temperature range 20–45°C, and ramping rate of 1°C/min). The gelation temperature of the bioinks was measured by observing the maximum point of *G*′ (*n* = 3).

The 3D structures and surface morphologies of the printed constructs were observed using a digital camera connected to an optical microscope (BX FM‐32; Olympus) and a scanning electron microscope (SEM) (SNE‐3000 M; SEC Inc.). Before visualizing the structures using SEM, samples were fixed with 10% neutral buffered formalin (NBF; Sigma‐Aldrich), dehydrated with an ethanol series (50%, 60%, 70%, 80%, 90%, and 100%), and then freeze‐dried.

To measure the tensile properties of the printed structures (5 × 15 × 1 mm^3^), a SurTA universal testing machine (Chemilab, South Korea) was used in the tensile testing mode (stretching rate: 0.05 mm/s). The Young's modulus was measured using the linear part (5%–10% strain) of the curves. All values are presented as a mean ± SD (*n* = 4). In addition, for the three types of MTJ units (2 × 6.5 × 1 mm^3^), the load–displacement curves were plotted after culturing for 1, 28, and 42 days. The elastic stiffness was evaluated using the slope of the linear part (~1 mm displacement) of the load–displacement curves (*n* < 4).

### 2.4 In vitro cellular responses

To analyze the cell viability after the fabrication process, the cells were stained with calcein AM (0.15 mM; Invitrogen) and ethidium homodimer 1 (2 mM; Invitrogen) for 1 h at 37°C. A confocal microscope was used to visualize the stained live (green) and dead (red) cells. Cell viability was estimated using ImageJ software (National Institutes of Health) by calculating the number of live cells per whole stained cell (*n* = 4).

To observe the morphologies of the cultured hASCs in the 3D structures, the nuclei and actin filaments (F‐actin) of the hASCs were observed. The cultured structures were fixed with 10% NBF for 30 min and permeabilized with 0.1% Triton X‐100 (Sigma‐Aldrich) for 10 min at 37°C. Then, the samples were incubated with 4′6‐diamidino‐2‐phenylindole (DAPI) (1:100 in DPBS; Invitrogen) and fluorescent phalloidin (1:100 in DPBS; Invitrogen) for 1 h at 37°C. The stained nuclei (blue) and F‐actin (green) of the cells were observed under a confocal microscope. The orientation factor (*f*) of the captured actin filaments was estimated using the ImageJ software (*n* = 4).

### 2.5 Immunofluorescence

The native and decellularized tissues and hASCs, seeded on the culture plates and contained in the bioinks and bioprinted 3D structures, were rinsed three times with DPBS and fixed using 10% NBF (Sigma‐Aldrich) for 60 min. The cells were then blocked and permeabilized using bovine serum albumin (2% BSA; Sigma‐Aldrich) and Triton X‐100 (2%; Sigma‐Aldrich). Primary antibodies, such as anti‐mouse collagen‐I primary antibody (5 μg/ml; Invitrogen), anti‐rabbit tenomodulin (TNMD) primary antibody (5 μg/ml; Invitrogen), anti‐mouse MF20 primary antibody (5 μg/ml; Developmental Studies Hybridoma Bank), anti‐mouse laminin‐α_1_ (LAMA1) primary antibody (5 μg/ml; Invitrogen), anti‐mouse scleraxis (SCX) primary antibody (5 μg/ml; Invitrogen), and anti‐rat integrin‐β1 primary antibody (5 μg/ml; Invitrogen), were used to observe cellular responses. After rinsing twice with DPBS, the samples were stained with Alexa Fluor 488 or Alexa Fluor 594‐conjugated anti‐mouse, anti‐rabbit, or anti‐rat secondary antibodies (1:50 in DPBS; Invitrogen) for 60 min, and counterstained with DAPI (5 μM in DPBS). The stained hASCs were visualized using a Carl Zeiss confocal microscope. The integrin‐β1positive area, myotube thickness, and TNMD positive area of the obtained images were quantitatively evaluated using ImageJ software (*n* = 4 or 5).

### 2.6 Real‐time polymerase chain reaction

To measure the gene expression levels, real‐time polymerase chain reaction (qRT‐PCR) was performed on cultured hASCs. The samples were treated with TRIzol reagent (Sigma‐Aldrich) to isolate the total RNA, followed by measuring the purity and concentration of the isolated RNA using a spectrophotometer (FLX800T; Biotek). cDNA was synthesized using RNase‐freeDNase‐treated total RNA and ReverTraAceqPCR RT Master Mix (Toyobo Co., Ltd.). Finally, a StepOnePlus Real‐Time PCR System (Applied Biosystems) and Thunderbird® SYBER® qPCR Mix (Toyobo Co., Ltd.) were used to perform qRT‐PCR. The measured expression levels were normalized to the housekeeping gene glyceraldehyde 3‐phosphate dehydrogenase. The gene expression levels of the hASCs seeded on the collagen‐coated culture plates, hASC‐containing collagen bioink, hASC‐containing collagen structure, and type 1 MTJ models were set to onefold. Table 1 lists the gene‐specific primers (Bionics) (*n* = 4).

**TABLE 1:** Primer sequences used in RT‐qPCR

Gene	Source	Primer sequence		GeneBank number
		Left (5′–3′)	Right (5′–3′)	
*Gapdh*	*Homo sapiens*	CCATGGGGAAGGTGAAGGTC	AGTGATGGCATGGACTGT	NM_002046.7
*Scx*	*Homo sapiens*	ACACCCAGCCCAAACAGAT	GCGGTCCTTGCTCAACTTT	NM_001080514.3
*Tnmd*	*Homo sapiens*	TGTATTGGATCAATCCCACTCTAAT	TTTTTCGTTGGCAGGAAAGT	NM_0022144.3
*Pax7*	*Homo sapiens*	TCAGGTGATGAGCATCTTGG	GCGGGGAGATGGAGAAGT	NM_013945.3
*Myf5*	*Homo sapiens*	CTATAGCCTGCCGGGACA	TGGACCAGACAGGACTGTTACAT	NM_005593.3
*Myod1*	*Homo sapiens*	CACTACAGCGGCGACTCC	TAGGCGCCTTCGTAGCAG	NM_002478.5
*Myh2*	*Homo sapiens*	GGTCTTGGACATTGCTGGTT	TTCTCATTGGTGAAGTTGATG	NM_017534.6
*Adipo*	*Homo sapiens*	GCTCTGTGCTCCTGCATCT	TTACGCTCTCCTTCCCCATA	NM_001177800.2
*Opn*	*Homo sapiens*	AAGTTTCGCAGACCTGACATC	GGGCTGTCCCAATCAGAAGG	NM_000582.2
*Acan*	*Homo sapiens*	AAGCACTGGAGTTCTGTGAATCT	CGGCATAGCACTTGTCCAG	NM_013227.3
*Neurod*	*Homo sapiens*	CTGCTCAGGACCTACTAACAACAA	GTCCAGCTTGGAGGACCTT	NM_002500.4
*Pxn*	*Homo sapiens*	TCGCTGTCGGATTTCAAGAT	ATGAACCCTCCCTCGTCCT	NM_001080855.3
*Tln1*	*Homo sapiens*	CGTGCAAACCAGGCAATTCA	CTCTTGGACTGCAGTGAGCA	NM_006289.4
*Thbs1*	*Homo sapiens*	ACACCGAAAGGGACGATGAC	AGTCAGGCACCCATAAGCTC	NM_003246.4
*Col1a1*	*Homo sapiens*	TGACGAGACCAAGAACTGCC	GCACCATCATTTCCACGAGC	NM_000088.4
*Lama1*	*Homo sapiens*	TGCTTCTGCTTTGGCGTTTC	TCCATCCACGCGGTAATAGC	NM_005559.4

Abbreviations: *GAPDH*: glyceraldehyde 3‐phosphate dehydrogenase; *Scx*: scleraxis; *Tnmd*: tenomodulin; *Pax7*: paired box 7; *Myf5*: myogenic factor 5; *Myod1*: myogenic differentiation 1; *Myh2*: myosin heavy chain 2; *Adipo*: adiponectin, C1Q, and collagen domain containing Adipoq; *Opn*: bone sialoprotein I (*Spp1*); *Acan*, aggrecan; *Neurod*, neuronal differentiation 1; *Pxn*, paxillin; *Tln1*, talin 1; *Thbs1*, thrombospondin 1; *Col1a1*, collagen type 1 alpha 1; *Lama1*, laminin alpha 1.

### 2.7 Statistical analyses

Statistical analyses were performed using SPSS software (SPSS, Inc.), conducted with the Student's *t*‐test (comparing two groups) and single‐factor ANOVA supplemented with Tukey's HSD post hoc test (comparing three or more groups). All quantitatively evaluated values are expressed as a mean ± SD, and values of p* < 0.05, p** < 0.01, and p*** < 0.001 were considered statistically significant.

## 3 RESULTS AND DISCUSSION

3D bioprinting has been widely used to fabricate micro‐ or macroscale complex structures by manipulating cells containing hydrogels into the required positions. However, the low mechanical properties of cell‐containing structures have been one of the overwhelming issues to be overcome in order to obtain a stable complex cell‐containing structure. To improve the poor mechanical properties, different mechanical supporting materials, such as graphene oxide,^26^ nanocellulose,^27^ methylcellulose,^28^ and structural reinforcing methods using synthetic polymers^13^, ^29^, ^30^, ^31^ have been used. Most methods have realized enhanced mechanical properties of the cell‐containing structures with minimal loss of cellular activity. However, a complex fabrication procedure and the cautious selection of biocompatible dispersed materials in hydrogels should be performed.

**FIGURE 1:**
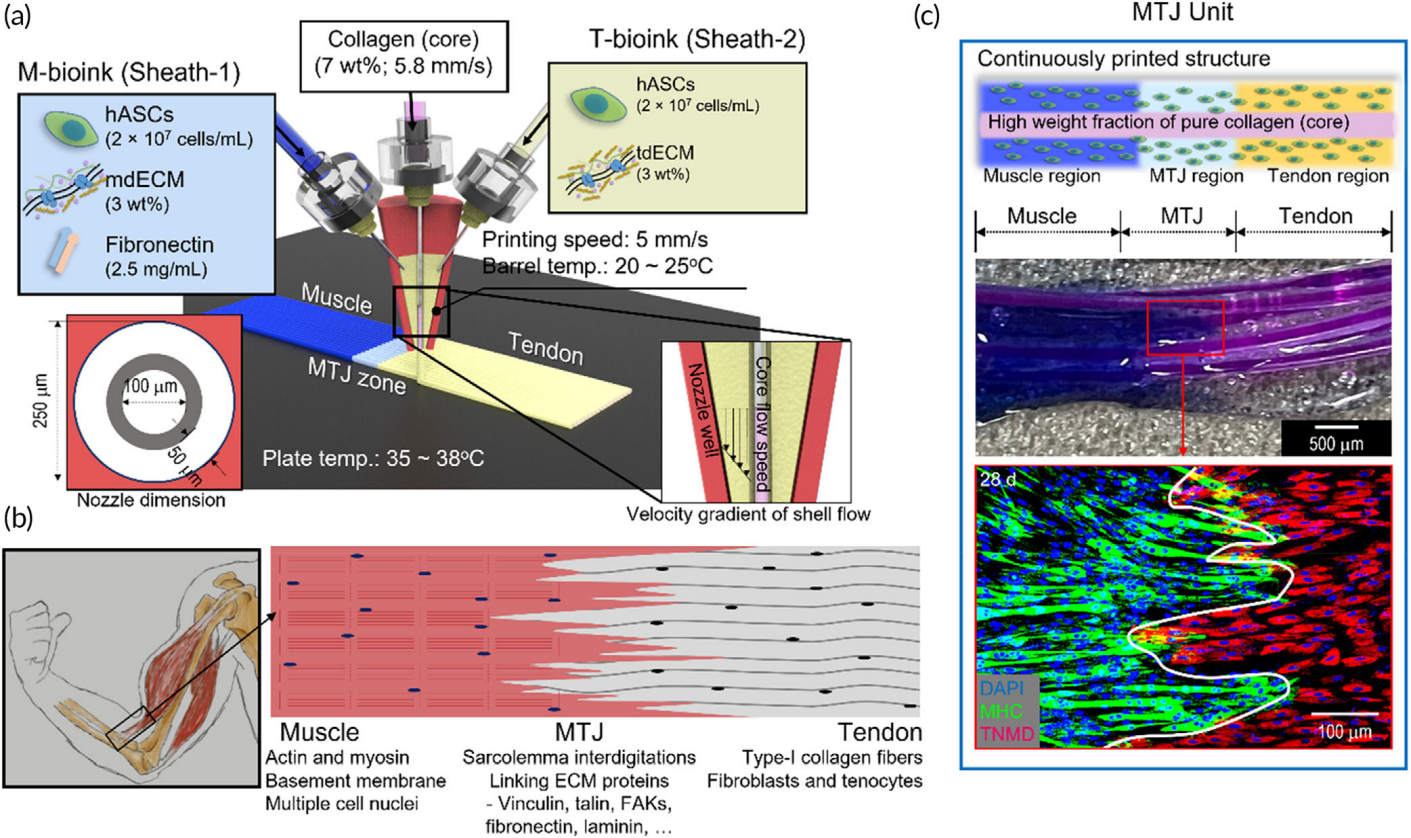
(a) Schematic of fabrication of myotendinous junction (MTJ) unit using a 3D bioprinting system with a modified core/sheath nozzle with a single core (collagen) and double sheath (M‐ and T‐bioink). (b) MTJ unit (muscle, MTJ, and tendon). (c) Optical and DAPI (blue)/myosin heavy chain (MHC; green)/tenomodulin (TNMD; red) (at 28 days) images of the bioprinted MTJ unit

In this study, we used a mechanically enhanced cell‐containing complex structure that can be applied to cell‐containing hydrogels by using a modified core–sheath nozzle for the 3D bioprinting. To fabricate the structure, we designed a core–sheath nozzle in which single‐ and double‐sheath channels were connected to the bioprinter (Figure 1a). A high weight fraction of collagen was fed to the core channel to enhance the mechanical properties of the cell‐containing structure, while the sheath channel carried muscle‐inducing bioink (M‐bioink; mdECM+fibronectin (2.5 mg/ml) and tendon‐inducing bioink (T‐bioink; tdECM) containing hASCs separately and simultaneously. In the M‐bioink, fibronectin is used to promote cellular proliferation and the primary state of myogenic differentiation because it is known to induce myofiber remodeling by attracting satellite and stem cells upon muscle injury.^32^, ^33^, ^34^ By controlling the ratio of the core (collagen) and sheath (M‐bioink and T‐bioink) flow rates, a complex tissue consisting of aligned muscle/tendon and myotendinous *junction* structures, as shown in the schematic in Figure 1b, could be obtained. Figure 1c shows optical and immunofluorescence images (DAPI: blue, MHC: green, and TNMD: red) of the bioprinted MTJ unit at 28 days.

### 3.1 Preparation of bioinks for MTJ unit

**FIGURE 2:**
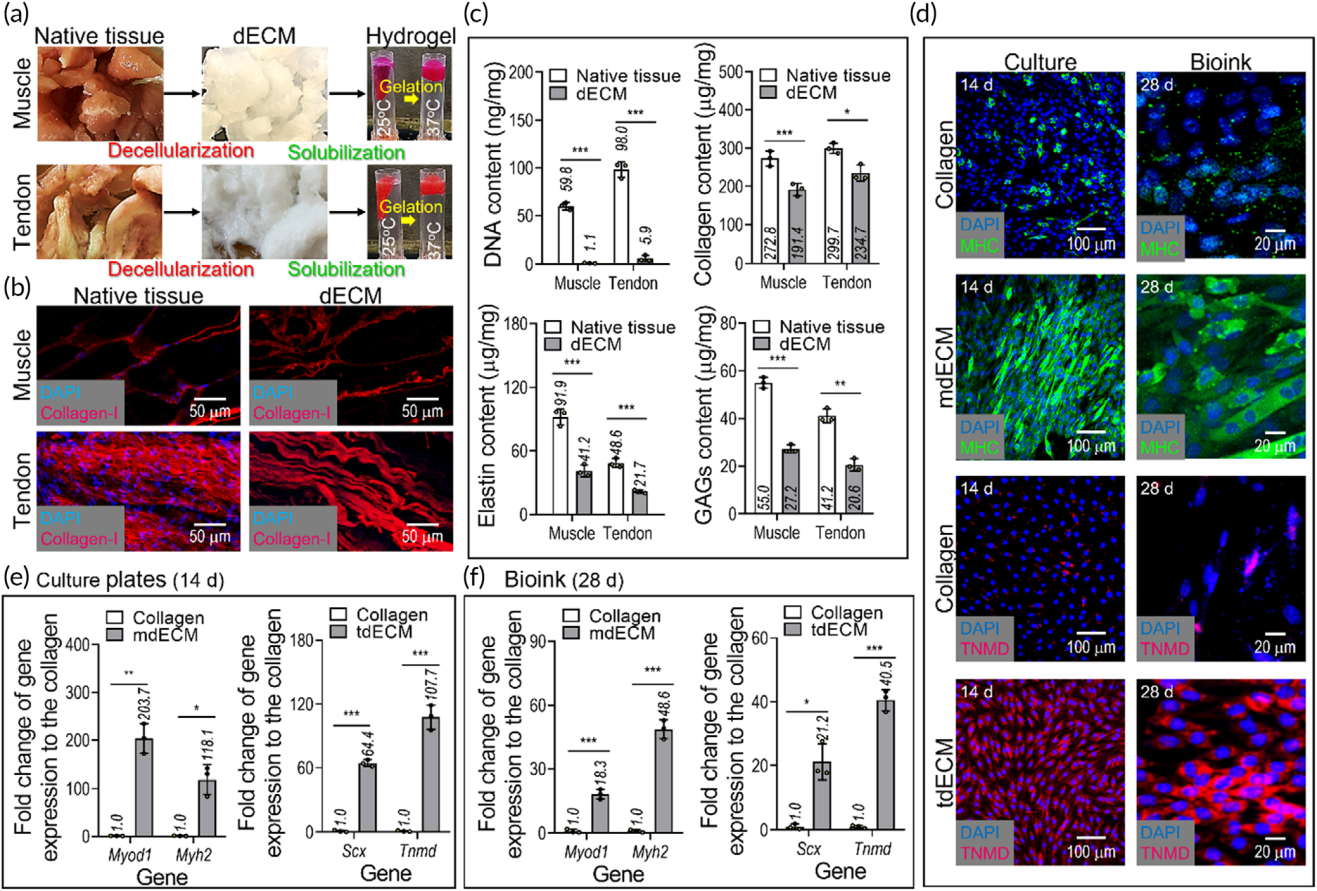
(a) Optical images showing native muscle/tendon tissues, decellularized extracellular matrices (dECMs), and their temperature‐dependent flowability. (b) 4′6‐diamidino‐2‐phenylindole (DAPI) (blue)/type I collagen (red) images of native and decellularized tissues. (c) DNA, collagen, elastin, and glycosaminoglycans (GAGs) contents of the native tissues, muscle‐derived dECM (mdECM), and tendon‐derived dECM (tdECM). (d) DAPI/TNMD (red) and DAPI/MHC (green) images of hASCs cultured on the tissue culture plates at 14 days, which were coated with collagen, mdECM, and tdECM, and laden in the bioinks (collagen, mdECM, and tdECM) at 28 days. Gene expression of myogenesis (*Myod1* and *Myh2*) and tenogenesis (*Scx* and *Tnmd*) of hASCs, cultured (e) on 2D culture plates and (f) in the bioinks (*n* = 3; *p < 0.050, **p < 0.010, and ***p < 0.001)

It has been well established that dECMs have unique biochemical/physiological signals showing the native microenvironments, and thus they have been used as functional and bioactive materials.^35^, ^36^, ^37^ To successfully create porcine‐ or bovine‐derived dECMs, bioactive constituents such as collagen, elastin, GAGs, and intricate biomolecules should be present, but the cell components should be completely removed.^37^ Here, we obtained dECMs derived from porcine skeletal muscle and Achilles tendon. As shown in the optical/immunofluorescence images (Figure 2a,b) and the different components (DNA content, collagen, elastin, and GAGs in Figure 2c) of the native tendon and muscle and each dECM, the key ECM components, collagen, elastin, and GAGs, were clearly present, whereas the cell components of the dECMs were completely removed by the decellularization process. In particular, the DNA contents were below 1.1 and 5.8 ng/mg (muscle and tendon, respectively) in terms of dry weight, indicating that the decellularization was satisfactory because any value less than 50 ng/mg can be regarded as being a suitable value for the DNA content of dECM.^38^

To show the bioactive feasibilities of the muscle and tendon dECMs, hASCs, specifically, mesenchymal stem cells derived from human adipose tissue were selected because they can be potentially differentiated into osteogenic, chondrogenic, myogenic, and even tenogenic lineages, by using biochemical, topographical, mechanical, and electrical stimulations.^39^ In particular, because the differentiation of hASCs can be directly affected by biochemical components, the myogenic and tenogenic differentiation of hASCs was evaluated by staining with MHC for muscle and TNMD for tendon.

The induction of myogenesis and tenogenesis by dECMs (mdECM: muscle‐derived dECM and tdECM: tendon‐derived dECM) on dECM‐coated tissue‐culture plates (2D culture) and hASC‐containing dECM bioinks (3D culture) were assessed in the absence of each differentiation medium, as shown in Figure 2d. The myogenic genes (*Myod1* and *Myh2*) and tenogenic (*Scx* and *TNMD*) were also measured after cell‐culturing for 14 and 28 days (tissue‐culture plate and bioink, respectively) (Figure 2e). As shown in the immunofluorescence images and gene expression results, the myogenic and tenogenic differentiation of the hASCs was clearly observed for the 2D and 3D cultures; in contrast, in the control material (type I collagen), the differentiation of the hASCs was very low. These results were in good agreement with those of previous studies.^40^, ^41^ Those studies revealed that the hASCs containing muscle‐derived dECM were fully differentiated into the myogenic lineage due to the biochemical components, transforming growth factor β1, insulin‐like growth factor 1, and vascular endothelial growth factor, among others.^24^, ^25^, ^42^, ^43^ In addition, the tenogenic differentiation of hASCs could be clearly observed due to the increase in several integrin subunits and TGF‐β/SMAD pathways in the hASCs, compared to the collagen control.^42^

### 3.2 Muscle and tendon structures bioprinted using dECMs and biophysical cues

**FIGURE 3:**
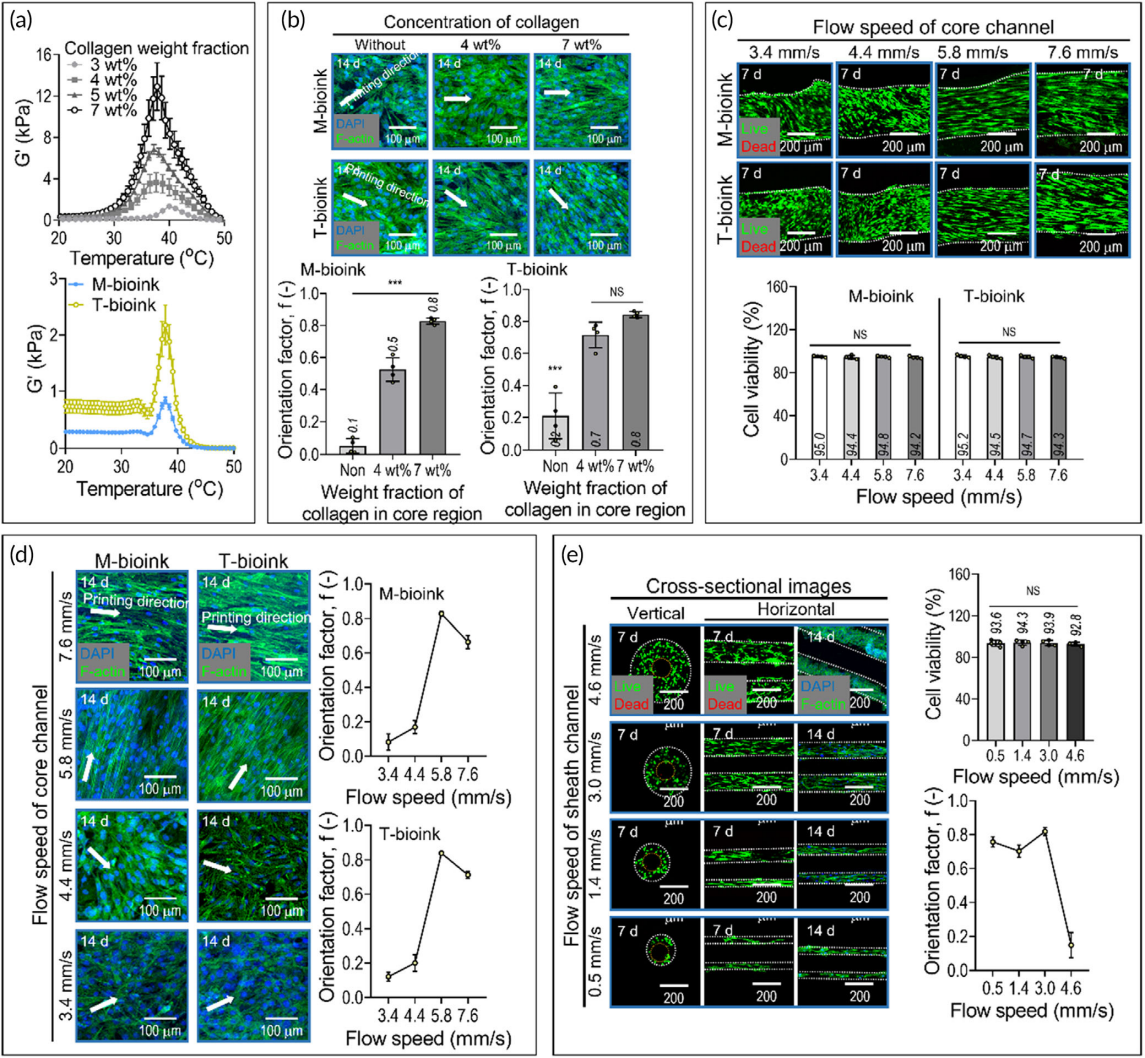
(a) Storage modulus (G') for different collagen concentrations (3, 4, 5, and 7 wt%) and two bioinks [M‐bioink: Hascs (2 × 10^7^ cells/ml) + 3 wt% mdECM + 2.5 mg/ml fibronectin, T‐bioink: 3 Wt% tdECM] for temperature sweep. (b) 4′6‐diamidino‐2‐phenylindole (DAPI)/phalloidin images of cells in the sheath were fabricated using the two bioinks with different collagen concentrations (0, 4, and 7 wt%) in the core and their orientation factors. (c) Live/dead images and cell‐viability (at 7 days) and (d) DAPI/phalloidin images and orientation factor, calculated using the F‐Actin, (at 14 days) of the sheath constructs fabricated using different flow rates of the collagen solution in the core. (e) Cross‐sectional live/dead images (7 days) and DAPI/phalloidin images (14 days) of the sheath construct were fabricated using different flow rates of the M‐bioink in the sheath (*n* = 4; ***p < 0.001)

The bioprinting process with a single core channel and double sheath channels was used to supply the pure collagen into the core region, and the hASCs containing M‐bioink and T‐bioink into the sheath regions. By controlling the flow rate of the two bioinks in the core and sheath nozzles, muscle, tendon, and MTJ regions were continuously fabricated. Because the rheological properties of the collagen and cell‐containing bioinks are different with respect to temperature, we set the barrel/nozzle temperature to 20–25°C of the collagen and the two dECM bioinks to avoid high wall shear stress within a sheath nozzle, affecting cell viability of the bioprinted structure.^44^ Furthermore, to maintain the physical shape of the bioprinted core–sheath structure, we set the temperature of the printing plate as the gelation temperature (35–38°C), as shown in Figure 3a.

In muscle and tendon tissue regeneration, highly aligned myoblasts, or tenocytes, which induce efficient myogenesis or tenogenesis, are key factors. Hence, we used a flow‐induced alignment of the cells in the bioinks by controlling (1) the collagen concentration in the core and (2) the flow rates in the core and sheath.

Figure 3b shows the effect of collagen concentration in the core on the alignment of the hASCs in the sheath. In the experiment, the flow rate of the collagen in the core and cell‐containing dECM bioinks was fixed to 5.8 and 3.0 mm/s, respectively. The results of live (green)/dead (red) (Figure S1) and DAPI (blue)/phalloidin (red) images of the cells in the bioinks processed with different collagen weight factions (0, 4, and 7 wt%) in the core, the relatively high weight fraction (7 wt%) of collagen in the core encouraged much higher F‐actin alignment (*f*), *f* = 0.8 ± 0.02 for M‐bioink and 0.8 ± 0.02 for T‐bioink, compared to those with no collagen flow in the core (*f* = 0.05 ± 0.1 for M‐bioink and 0.21 ± 0.1 for T‐bioink) and 4 wt% of collagen (*f* = 0.5 ± 0.1 for M‐bioink and 0.7 ± 0.1 for T‐bioink) (Figure 3b). The orientation factor (*f*) was calculated using the equation, *f* = (90° − *ψ*)/90°, where *ψ* is the full width at half maximum of the distribution curve of F‐actin to the printing direction, and the values, 0 and 1, indicate random distribution and full alignment, respectively.^44^ We believe that the cell alignment in the bioinks in the sheath may be different due to the rheological properties of the collagen flowing through the core. In particular, for low‐viscosity collagen, the cells in the bioinks in the sheath cannot be fully aligned owing to the low friction force between the collagen and the bioink in the sheath, whereas the flow of highly viscous collagen can be expected to increase the cell alignment by applying a relatively higher friction force to the cells of bioinks in the sheath. For this reason, we fixed the weight fraction (7 wt%) of the collagen flowing in the core.

In addition, to observe the effects of the flow rates of the collagen in the core on the cell alignment in the sheath, we varied the flow rate of the collagen in the core (Figure 3c,d). As the flow rate of the collagen is increased, there should be a greater friction force between the collagen solution in the core and the bioinks in the sheath. After printing the T‐ and M‐bioinks (at a fixed flow rate of 3.0 mm/s) in the sheath and printing the collagen (7 wt%) with a range of flow rates (3.4–7.6 mm/s) in the core, the live/dead ratio at 7 days and DAPI/phalloidin images at 14 days were obtained. As shown in the live/dead images, the cells in the bioinks were viable (cell viability: ~ 90%). The orientation factor was measured at different core collagen flow rates to quantitatively assess the alignment of the cells in the sheath. As expected, the cell alignment in the bioinks flowing in the sheath was clearly improved with an increase in the collagen flow rate. However, a comparatively higher flow rate (7.6 mm/s) of collagen in the core can cause a slight disturbance in the cell alignment.

We also observed the effect of the flow rate in the sheath on the cell alignment. In the test, the collagen flow rate in the core was fixed to 5.8 mm/s, and the flow rate of the M‐bioink in the sheath was varied (0.5–4.6 mm/s). As shown in the cross‐sectional live/dead images (7 days) and DAPI/phalloidin images (14 days) in Figure 3e, the diameter of the sheath increased with the flow rate of the bioink, while the diameter of the core carrying the collagen remained steady due to the constant flow rate. Moreover, the cells in the sheath were alive across the entire range of bioink flow rates. Regarding the orientation factor, the aligned F‐actin of the cells was well developed for most flow rates. However, as the flow rate of the bioink in the sheath exceeded 4.6 mm/s, the aligned morphology of the cells was not well developed because of the comparatively high‐volume flow rate of the bioink, thus increasing the high extrudate‐swelling inducing the randomness of the cell alignment.

Based on the analysis of the printing parameters, we selected the flow rates in the core and sheath of 5.8 and 3.0 mm/s in the core and sheath, respectively.

### 3.3 Myogenic and tenogenic differentiation of hASCs in the bioinks

**FIGURE 4:**
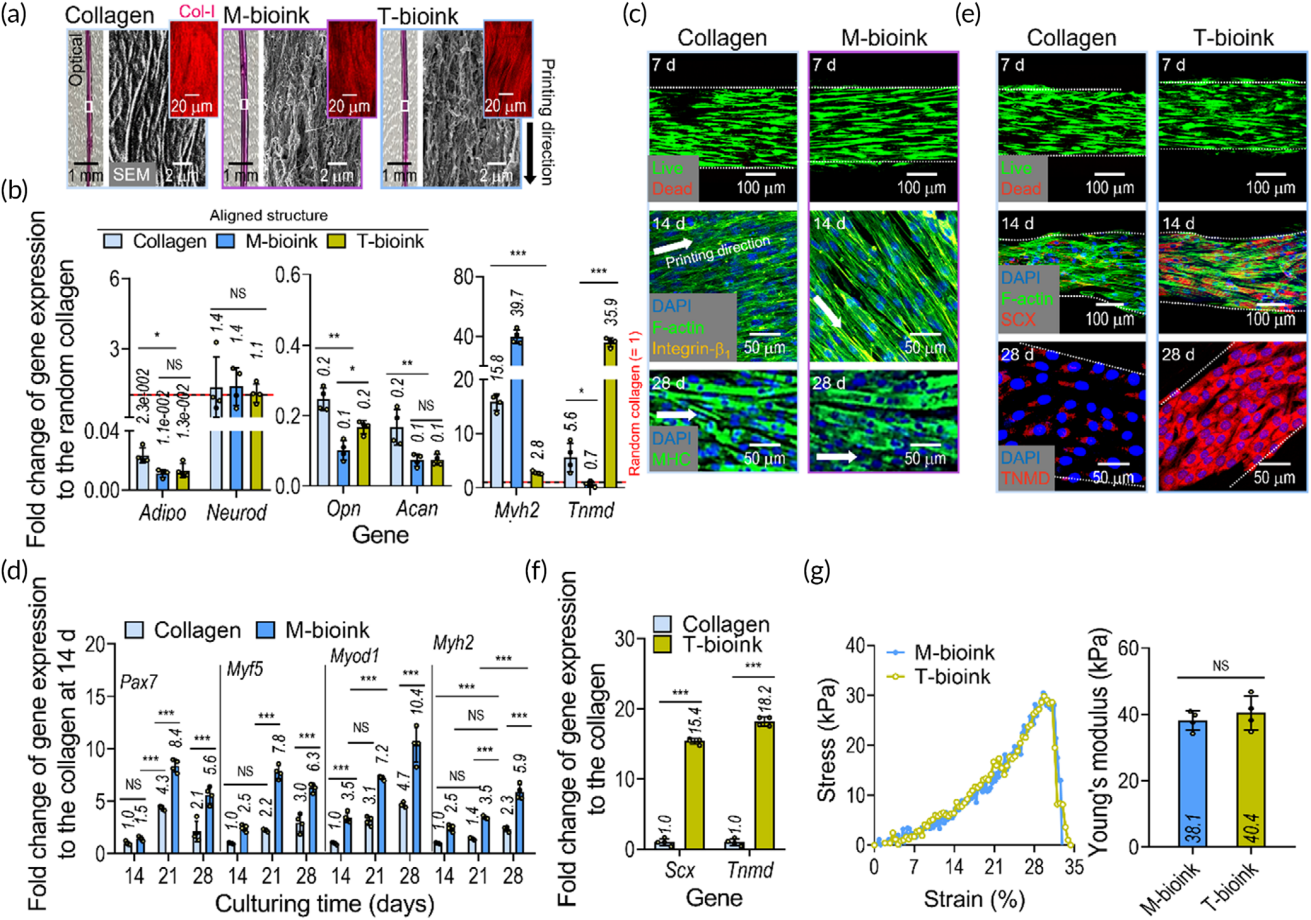
(a) Optical, scanning electron microscope, and in situ type‐I collagen immunofluorescence (Col‐I; red) images of the fabricated constructs using hASCs‐containing collagen, M‐bioink, and T‐bioink. (b) Gene expression result of *Adipo*, *Opn*, *Acan*, *Neurod*, *Myh2*, and *Tnmd* for the bioprinted constructs (at 28 days). The expression levels were normalized to the results for the cultured hASCs‐containing collagen bioink (without printing). (c) Live/dead (at 7 days), 4′6‐diamidino‐2‐phenylindole (DAPI)/F‐actin/integrin‐β1 (yellow) (at 14 days), and DAPI/MHC (green) (at 28 days) images of the cell‐containing collagen construct (control) and M‐bioink. (d) Myogenesis‐related gene expression results, including *Pax7*, *Myf5*, *Myod1*, and *Myh2*, of the collagen control and M‐bioink at 28 days. (e) Live /dead (at 7 days), DAPI/F‐actin/scleraxis (SCX; red) (at 14 days), and DAPI/TNMD (red) (at 28 days) images of the collagen control and T‐bioink. (f) Expression levels of *Scx* and *Tnmd* for the collagen control and T‐bioink at 28 days. (g) Stress–strain curves and Young's modulus for the structures fabricated using M‐bioink and T‐bioink (*n* = 4; *p < 0.050, **p < 0.010, and ***p < 0.001)

To observe the differentiation ability of the hASCs in the M‐ and T‐bioinks, the expressions of the different genes in the bioinks were assessed. To fabricate the cell‐containing structures using the bioinks, printing parameters were used with the previously selected parameters. Figure 4a shows the fabricated constructs using cell‐containing collagen (control), cell‐containing M‐bioink, and cell‐containing T‐bioink. As shown in the morphological structure of the SEM and the type‐I collagen immunofluorescence (Col‐I) images, the aligned morphological structure in the fabricated cell‐containing structures could be clearly observed.

The bioprinted hASC‐containing constructs were cultured without using any differentiation medium for 28 days, and gene expression analysis was conducted. The mRNA levels of the musculoskeletal differentiation genes, such as the adipogenic, osteogenic, chondrogenic, neurogenic, myogenic (*Myh2*), and tenogenic genes were screened (Figure 4b). The attained genes were used to show whether the biochemical cue released from the bioinks and the alignment cue provided by the bioprinting process can encourage myogenic or tenogenic differentiation of hASCs cultured in growth medium alone, DMEM/low‐G, FBS, and penicillin–streptomycin. The results showed that the hASCs were well differentiated along the myogenic and tenogenic lineage due to the biochemical and biophysical cues, thus mimicking the microenvironmental conditions of the native muscle and tendon tissues, whereas the stem cells processed with the pure collagen bioink were differentiated into myogenic lineages within a low range due to biophysical cues,^24^ but did not fully differentiate into tenogenic lineages.

To illustrate the differentiation of stem cells into each myogenic or tenogenic lineage, the live/dead, DAPI/phalloidin/integrin‐β1, and MHC immunostaining images are shown in Figure 4c. As expected, the biochemical cues from the muscle‐derived dECM and physical cues induced by the 3D bioprinting process enhanced the degree of myogenesis (i.e., increased MHC thickness shown in the image) of the hASCs in the bioink, compared to the collagen control with only the alignment cue. The genes related to myogenesis (*Pax7*, *Myf5*, *Myod1*, and *Myh2*) of the cells in the M‐bioink and collagen are shown in Figure 4d. From these results, we confirmed that hASCs in the structure fabricated using M‐bioink were well differentiated into the myogenic lineage, relative to the collagen bioink.

In addition, to observe the tenogenic differentiation of the hASCs in the structure fabricated using the T‐bioink, we measured the live/dead, DAPI/phalloidin/SCX, TNMD immunostaining images, and gene expression (*Scx* and *Tnmd*) (Figure 4e,f). As shown in the results, the alignment of the F‐actin and the development of SCX and TNMD for the structure fabricated using T‐bioink was significantly greater than that for the collagen‐control bioink. We believe that the tenogenic differentiation of hASCs can be clearly supported by the biochemical components of tendon‐derived dECM, which induces an increase in several integrin subunits (TGF‐β and p‐SMAD2 in hASCs), which was well evaluated in a previous study.^42^ Furthermore, tenogenic differentiation can be dependent on mechanical stiffness. For example, as mesenchymal stem cells were cultured on a collagen scaffold, a stiffness of approximately 30–50 kPa can encourage tenogenic differentiation of the cells, whereas a much stiffer scaffold of around 70–90 kPa strongly encouraged osteogenic differentiation.^45^ In this aspect, we measured the stiffness of the printed structure, and we obtained the stiffness (approximately 40 ± 5 kPa) of the structure with topographical and biochemical cues (Figure 4g).

Thus, we can confirm that the stem cells in the constructs with biophysical cues, bioprinted using two bioinks (M‐ and T‐bioink) can clearly differentiate into myogenic and tenogenic lineages.

### 3.4 Fabrication of three MTJ constructs and their formation of an MTJ unit

**FIGURE 5:**
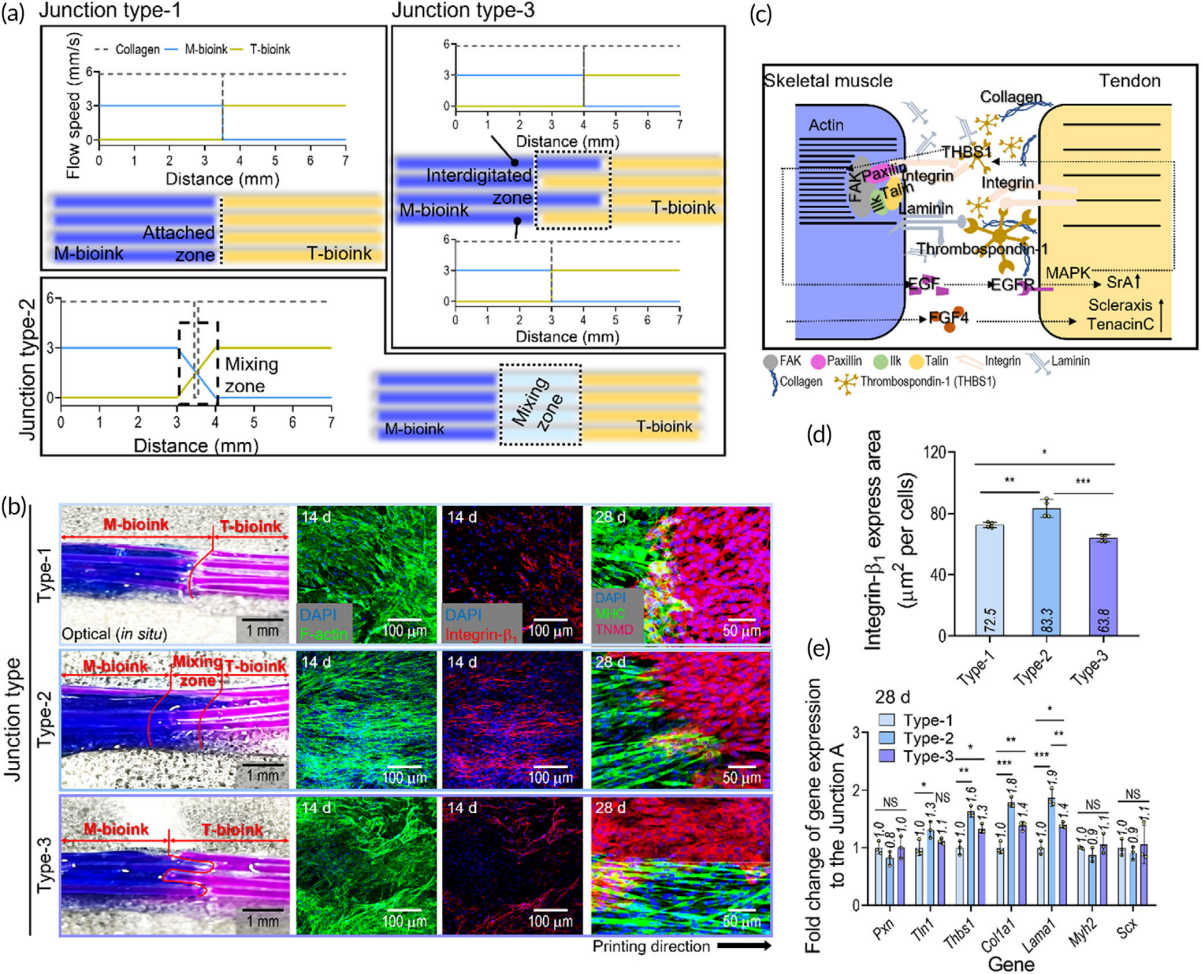
(a) Schematics showing the fabrication procedure for three types of myotendinous junction (MTJ) unit (type 1: Direct contact between muscle and tendon, type 2: Contact in mixing zone, and type 3: Interdigitated contact). (b) Optical, 4′6‐diamidino‐2‐phenylindole (DAPI)/F‐Actin (at 14 days), DAPI/integrin‐β1 (red) (at 14 days), DAPI/F‐Actin/integrin‐β1 (at 14 days), and DAPI/MHC/TNMD (at 28 days) images for the three types of MTJ interfaces. (c) Schematic showing the MTJ ECM composition and crosstalk between the muscle and tendon cells. (d) Level of integrin‐β1 expression using fluorescence images (*n* = 4). (e) Expression level of the MTJ‐associated genes (*Scx*, *Pxn*, *Tln1*, *Thbs1*, *Col1a1*, *Lama1*, and *Myh2*) for the three fabricated types of MTJ structure (*n* = 3) (*p < 0.050, **p < 0.010, and ***p < 0.001)

To obtain the three different interface models (type 1: direct contact between muscle and tendon cells; type 2: artificially designed mixed zone of muscle and tendon cells, type 3: interdigitated contact between the muscle and tendon cells, as shown in Figure 5a) of the MTJ unit, the M‐bioink was printed on one side and the T‐bioink on the other side by regulating the flow rates of each of the M‐ and T‐bioinks in the core–sheath nozzle, as shown in the schematic in Figure 5a.

In general, cell‐laden constructs could be biodegradable to be replaced with the newly formed tissues. Additionally, the rate of biodegradation for cell‐laden constructs can directly affect several cellular responses. To assess the biodegradability of the bioinks [the collagen (7 wt%), M‐bioink (3 wt%), and T‐bioink (3 wt%) without cells], the printed structures were incubated in collagenase solution (0.1 U/ml of DPBS).^25^ The biodegradation of the constructs measured over 21 days was presented in Figure S2A. In the results, the pure collagen was degraded at around 20.8%, but the M‐ and T‐bioinks were more rapidly degraded by about 1.6‐fold (M‐bioink 34.9% and T‐bioink 33.8%). In addition, we measured the biodegradability of the printed MTJ constructs without cells. As shown in Figure S2B, the degradation of the MTJ constructs (type 1 = 24.0%, type 2 = 24.8%, and type 3 = 24.3% at 21 days) was less than the constructs fabricated using cell‐free M‐ and T‐bioinks due to the relatively slow degradation of collagen laden in the core region of the MTJ constructs. Although the biodegradation of the dECM‐bioinks in the MTJ constructs was rapid, we carefully estimated that the ECM developed from the cells laden in the MTJ constructs could quickly recover the region of the degraded bioinks, and the collagen in the core region of the MTJ constructs could provide mechanical stability.

After the 14‐day cell culture, the optical and fluorescence images of the fabricated MTJ units were analyzed to observe the MTJ development for each model (Figure 5b). On each side of the MTJ structure, the hASCs were well differentiated into myogenic and tenogenic lineages according to the biochemical and biophysical cues. The MHCs on the muscle side in the MTJ constructs were well expressed with the aligned and multinucleated myotubes, while on the tendon side, TNMD was well expressed in the constructs. However, when comparing the MTJ formation of the three models, the type 2 interface exhibited a much more developed integrinβ1 expression and activated sarcolemma of the finger‐like process that was well anchored in the tendon, relative to the type 1 and 2 interfaces, with the pattern being more similar to that of a natural MTJ tissue interface.

Adhesive proteins, such as paxillin, integrin, fibronectin, and talin, have been found in the region of the MTJ to permit a connection between the aligned actin filaments and collagen fibers in tendons, as shown in the schematic image in Figure 5c.^5^, ^46^ To observe the completeness of MTJ development, we measured several genes for the three different types of MTJ constructs (Figure 5d,e). As shown in the integrin‐β1 and MTJ‐associated genes (*Pxn*, *Tln1*, *Thbs1*, *Col1a1*, *Lama1*, *Myh2*, and *Scx*), the type 2 MTJ construct exhibited a significantly higher gene expression than those of the type 1 and type 3 constructs. This result indicates that MTJ formation can be clearly affected by the physical interfacing shape between the muscle and tendon cells.

**FIGURE 6:**
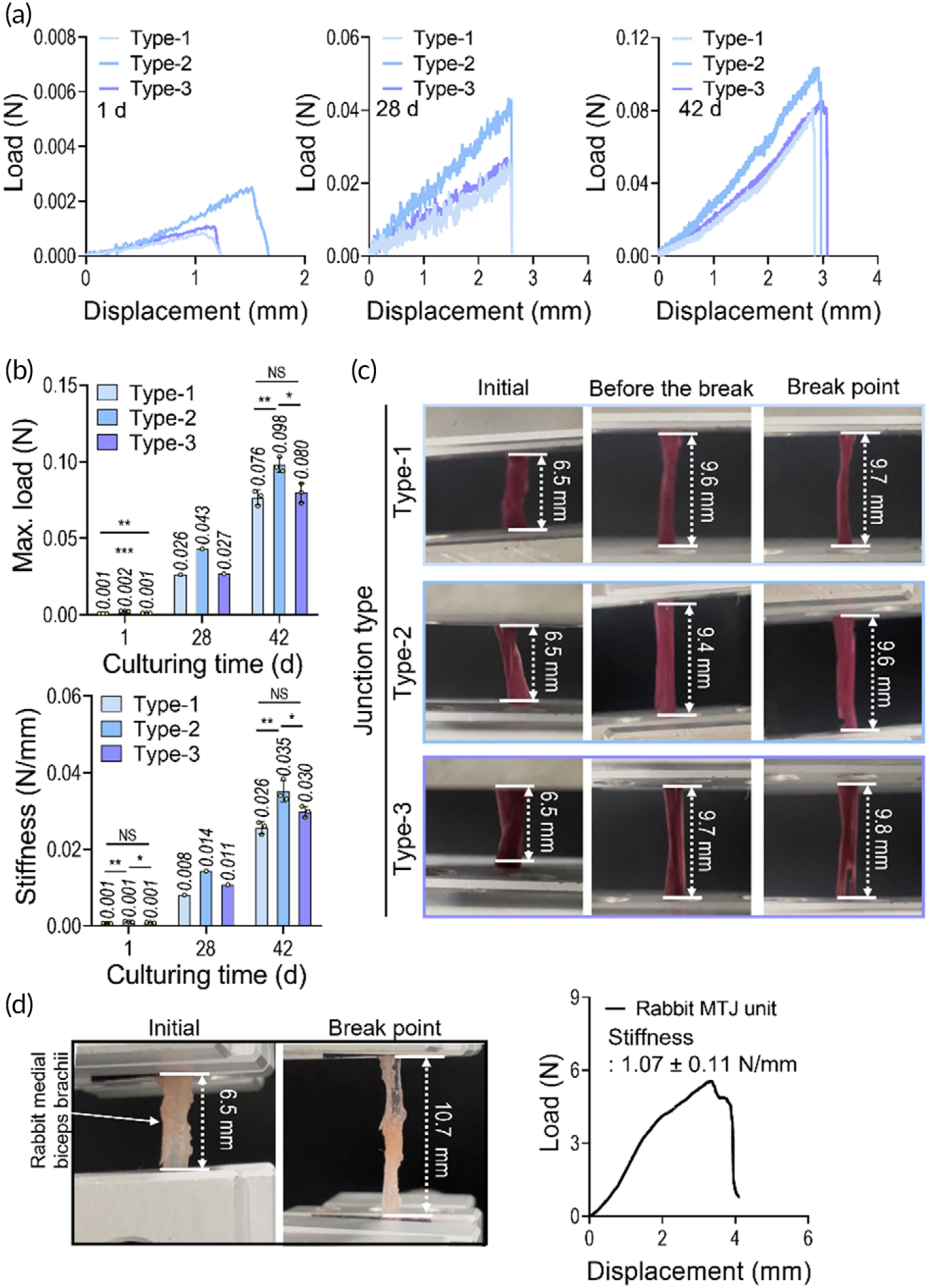
(a) Load–displacement curves for junction types 1, 2, and 3 at 1, 28, and 42 days of cell culture. (b) Maximum load and stiffness of myotendinous junction (MTJ) structures estimated using the load–displacement curves. (c) Captured images of the samples at initial and break point. (d) A load–displacement curve and stiffness of MTJ unit of a rabbit medial biceps brachii (*n* < 4, *p < 0.050, **p < 0.010, and ***p < 0.001)

After culturing the fabricated constructs, in situ and for 28 and 42 days, the mechanical properties of the bioprinted MTJ units were assessed. In Figure 6a–c, the force−displacement curves, maximum load and stiffness, and optical images showing the initial and break point of the samples calculated using 10% strain are shown. As shown in the results, as the culture period increased, the mechanical properties of all the MTJ units were enhanced, although the properties were significantly lower than those of the rabbit, shown in Figure 6d. However, when comparing the three MTJ units, the maximum load and elastic stiffness of the type 2 MTJ construct were meaningfully greater than those of the other types. This result can be well validated with the MTJ‐associated genes, shown in Figure 5e, because of the well‐developed ECM components released from the interaction between the cells during the cell culture period.

## 4 CONCLUSION

The objective of this study was to develop an in vitro 3D complex tissue structure resembling a native MTJ, fabricated using modified bioprinting supplemented with the bioinks of muscle‐ and tendon‐derived dECMs and hASCs. The dECM‐based bioinks and cell‐alignment cue attained with the optimal bioprinting condition effectively induced hASCs to myogenic and tenogenic differentiation. The fabricated muscle and tendon tissues exhibited well‐aligned morphological structures and highly developed biochemical characteristics of the muscle and tendon tissues. Three MTJ interface models were suggested with different physical interfacing shapes, and the type 2 interface using a mixing zone of muscle‐tendon cells exhibited a much more activated sarcolemma of the finger‐like process with significantly enhanced MTJ‐associated genes, compared to the other interfaces. Based on these results, we believe that the suggested in vitro 3D MTJ model can be useful for understanding how the specialized MTJ tissue interface can be developed and maintained.

## AUTHOR CONTRIBUTIONS

**Won Jin Kim:** Conceptualization (equal); data curation (equal); formal analysis (equal); investigation (equal); writing – original draft (equal). **Geun Hyung Kim:** Conceptualization (equal); data curation (equal); formal analysis (equal); funding acquisition (lead); investigation (equal); methodology (equal); resources (lead); supervision (lead); writing – review and editing (lead).

### PEER REVIEW

The peer review history for this article is available at https://publons.com/publon/10.1002/btm2.10321.

## Supporting information


**Appendix S1** Supporting Information

## Data Availability

The data that support the findings of this study are available from the corresponding author upon reasonable request.

## References

[1] Phillips JE , Burns KL , Le Doux JM , Guldberg RE , García AJ . Engineering graded tissue interfaces. Proc Natl Acad Sci USA. 2008;105:12170‐12175. 18719120 10.1073/pnas.0801988105 PMC2527884

[2] Mahjoub M , Berenbaum F , Houard X . Why subchondral bone in osteoarthritis? The importance of the cartilage bone interface in osteoarthritis. Osteoporos Int. 2012;23:841‐846. 23179566 10.1007/s00198-012-2161-0

[3] Baldino L , Cardea S , Maffulli N , Reverchon E . Regeneration techniques for bone‐to‐tendon and muscle‐to‐tendon interfaces reconstruction. Br Med Bull. 2016;117:25‐37. 26837850 10.1093/bmb/ldv056

[4] Tellado SF , Balmayor ER , Van Griensven M . Strategies to engineer tendon/ligament‐to‐bone interface: biomaterials, cells and growth factors. Adv Drug Deliv Rev. 2015;94:126‐140. 25777059 10.1016/j.addr.2015.03.004

[5] Bayrak E , Yilgor HP . Engineering musculoskeletal tissue interfaces. Front Mater. 2018;5:24.

[6] Lysholm J , Wiklander J . Injuries in runners. Am J Sports Med. 1987;15:168‐171. 3578639 10.1177/036354658701500213

[7] VanDusen K , Larkin L . Muscle–tendon interface. In: Nukavarapu SP , Freeman JW , Laurencin CT , eds. Regenerative Engineering of Musculoskeletal Tissues and Interfaces. Woodhead Publishing; 2015:409‐429.

[8] Galatz LM , Ball CM , Teefey SA , Middleton WD , Yamaguchi K . The outcome and repair integrity of completely arthroscopically repaired large and massive rotator cuff tears. J Bone Jt Surg. 2004;86:219‐224. 10.2106/00004623-200402000-00002 14960664

[9] Vitale MA , Vitale MG , Zivin JG , Braman JP , Bigliani LU , Flatow EL . Rotator cuff repair: an analysis of utility scores and cost‐effectiveness. J Shoulder Elbow Surg. 2007;16:181‐187. 17399623 10.1016/j.jse.2006.06.013

[10] Sato EJ , Killian ML , Choi AJ , et al. Skeletal muscle fibrosis and stiffness increase after rotator cuff tendon injury and neuromuscular compromise in a rat model. J Orthopaed Res. 2014;32:1111‐1116. 10.1002/jor.22646 PMC4415493 24838823

[11] Ladd MR , Lee SJ , Stitzel JD , Atala A , Yoo JJ . Co‐electrospun dual scaffolding system with potential for muscle–tendon junction tissue engineering. Biomaterials. 2011;32:1549‐1559. 21093046 10.1016/j.biomaterials.2010.10.038

[12] Larkin LM , Calve S , Kostrominova TY , Arruda EM . Structure and functional evaluation of tendon–skeletal muscle constructs engineered in vitro. Tissue Eng. 2006;12:3149‐3158. 17518629 10.1089/ten.2006.12.3149 PMC2798802

[13] Merceron TK , Burt M , Seol Y‐J , et al. A 3D bioprinted complex structure for engineering the muscle–tendon unit. Biofabrication. 2015;7:035003. 26081669 10.1088/1758-5090/7/3/035003

[14] Koeck KS , Salehi S , Humenik M , Scheibel T . Processing of continuous non‐crosslinked collagen fibers for microtissue formation at the muscle‐tendon Interface. Adv Funct Mater. 2021;2112238:2112238.

[15] Shiroud Heidari B , Ruan R , De‐Juan‐Pardo EM , Zheng M , Doyle B . Biofabrication and signaling strategies for tendon/ligament interfacial tissue engineering. ACS Biomater Sci Eng. 2021;7:383‐399. 33492125 10.1021/acsbiomaterials.0c00731

[16] Balestri W , Morris RH , Hunt JA , Reinwald Y . Current advances on the regeneration of musculoskeletal interfaces. Tissue Eng Part B Rev. 2021;27:548‐571. 33176607 10.1089/ten.TEB.2020.0112

[17] Ovsianikov A , Khademhosseini A , Mironov V . The synergy of scaffold‐based and scaffold‐free tissue engineering strategies. Trends Biotechnol. 2018;36:348‐357. 29475621 10.1016/j.tibtech.2018.01.005

[18] Hutmacher DW , Sittinger M , Risbud MV . Scaffold‐based tissue engineering: rationale for computer‐aided design and solid free‐form fabrication systems. Trends Biotechnol. 2004;22:354‐362. 15245908 10.1016/j.tibtech.2004.05.005

[19] Juncosa‐Melvin N , Boivin GP , Galloway MT , Gooch C , West JR , Butler DL . Effects of cell‐to‐collagen ratio in stem cell‐seeded constructs for Achilles tendon repair. Tissue Eng. 2006;12:681‐689. 16674283 10.1089/ten.2006.12.681

[20] Turner NJ , Badylak SF . Biologic scaffolds for musculotendinous tissue repair. Eur Cell Mater. 2013;25:43. 10.22203/ecm.v025a09 23329468

[21] Laranjeira M , Domingues RMA , Costa‐Almeida R , Reis RL , Gomes ME . 3D mimicry of native‐tissue‐fiber architecture guides tendon‐derived cells and adipose stem cells into artificial tendon constructs. Small. 2017;13:1700689. 10.1002/smll.201700689 28631375

[22] Yang C , Deng G , Chen W , Ye X , Mo X . A novel electrospun‐aligned nanoyarn‐reinforced nanofibrous scaffold for tendon tissue engineering. Colloids Surf B Biointerfaces. 2014;122:270‐276. 25064476 10.1016/j.colsurfb.2014.06.061

[23] Zhao F , Cheng J , Zhang J , et al. Comparison of three different acidic solutions in tendon decellularized extracellular matrix bio‐ink fabrication for 3D cell printing. Acta Biomater. 2021;131:262‐275. 34157451 10.1016/j.actbio.2021.06.026

[24] Kim W , Lee H , Lee CK , et al. A bioprinting process supplemented with in situ electrical stimulation directly induces significant Myotube formation and Myogenesis. Adv Funct Mater. 2021;31:2105170.

[25] Kim W , Lee H , Lee J , et al. Efficient myotube formation in 3D bioprinted tissue construct by biochemical and topographical cues. Biomaterials. 2020;230:119632. 31761486 10.1016/j.biomaterials.2019.119632 PMC7141931

[26] Cha C , Shin SR , Gao X , et al. Controlling mechanical properties of cell‐laden hydrogels by covalent incorporation of graphene oxide. Small. 2014;10:514‐523. 24127350 10.1002/smll.201302182 PMC3946390

[27] Markstedt K , Mantas A , Tournier I , Martínez Ávila H , Hägg D , Gatenholm P . 3D bioprinting human chondrocytes with nanocellulose–alginate bioink for cartilage tissue engineering applications. Biomacromolecules. 2015;16:1489‐1496. 25806996 10.1021/acs.biomac.5b00188

[28] Li H , Tan YJ , Leong KF , Li L . 3D bioprinting of highly thixotropic alginate/methylcellulose hydrogel with strong interface bonding. ACS Appl Mater Interfaces. 2017;9:20086‐20097. 28530091 10.1021/acsami.7b04216

[29] Lee H , Ahn S , Bonassar LJ , Kim G . Cell (MC3T3‐E1)‐printed poly (ϵ‐caprolactone)/alginate hybrid scaffolds for tissue regeneration. Macromol Rapid Commun. 2013;34:142‐149. 23059986 10.1002/marc.201200524

[30] Kundu J , Shim JH , Jang J , Kim SW , Cho DW . An additive manufacturing‐based PCL–alginate–chondrocyte bioprinted scaffold for cartilage tissue engineering. J Tissue Eng Regen Med. 2015;9:1286‐1297. 23349081 10.1002/term.1682

[31] Unagolla JM , Jayasuriya AC . Hydrogel‐based 3D bioprinting: a comprehensive review on cell‐laden hydrogels, bioink formulations, and future perspectives. Appl Mater Today. 2020;18:100479. 32775607 10.1016/j.apmt.2019.100479 PMC7414424

[32] Schüler SC , Kirkpatrick JM , Schmidt M , et al. Extensive remodeling of the extracellular matrix during aging contributes to age‐dependent impairments of muscle stem cell functionality. Cell Rep. 2021;35:109223. 34107247 10.1016/j.celrep.2021.109223

[33] Garcia JMS , Panitch A , Calve S . Functionalization of hyaluronic acid hydrogels with ECM‐derived peptides to control myoblast behavior. Acta Biomater. 2019;84:169‐179. 30508655 10.1016/j.actbio.2018.11.030 PMC6326884

[34] Vaz R , Martins GG , Thorsteinsdóttir S , Rodrigues G . Fibronectin promotes migration, alignment and fusion in an in vitro myoblast cell model. Cell Tissue Res. 2012;348:569‐578. 22427060 10.1007/s00441-012-1364-1

[35] Choudhury D , Tun HW , Wang T , Naing MW . Organ‐derived decellularized extracellular matrix: a game changer for bioink manufacturing? Trends Biotechnol. 2018;36:787‐805. 29678431 10.1016/j.tibtech.2018.03.003

[36] Shakouri‐Motlagh A , O'Connor AJ , Brennecke SP , Kalionis B , Heath DE . Native and solubilized decellularized extracellular matrix: a critical assessment of their potential for improving the expansion of mesenchymal stem cells. Acta Biomater. 2017;55:1‐12. 28412553 10.1016/j.actbio.2017.04.014

[37] Hussey GS , Dziki JL , Badylak SF . Extracellular matrix‐based materials for regenerative medicine. Nat Rev Mater. 2018;3:159‐173.

[38] Crapo PM , Gilbert TW , Badylak SF . An overview of tissue and whole organ decellularization processes. Biomaterials. 2011;32:3233‐3243. 21296410 10.1016/j.biomaterials.2011.01.057 PMC3084613

[39] Augello A , De Bari C . The regulation of differentiation in mesenchymal stem cells. Hum Gene Ther. 2010;21:1226‐1238. 20804388 10.1089/hum.2010.173

[40] Kim W , Jang CH , Kim G . Bioprinted hASC‐laden structures with cell‐differentiation niches for muscle regeneration. Chem Eng J. 2021;419:129570.

[41] Wang D , Pun CC , Huang S , et al. Tendon‐derived extracellular matrix induces mesenchymal stem cell tenogenesis via an integrin/transforming growth factor‐β crosstalk‐mediated mechanism. FASEB J. 2020;34:8172‐8186. 32301551 10.1096/fj.201902377RR

[42] Wang L , Johnson JA , Chang DW , Zhang Q . Decellularized musculofascial extracellular matrix for tissue engineering. Biomaterials. 2013;34:2641‐2654. 23347834 10.1016/j.biomaterials.2012.12.048 PMC4801146

[43] Yi H , Forsythe S , He Y , et al. Tissue‐specific extracellular matrix promotes myogenic differentiation of human muscle progenitor cells on gelatin and heparin conjugated alginate hydrogels. Acta Biomater. 2017;62:222‐233. 28823716 10.1016/j.actbio.2017.08.022 PMC8151673

[44] Kim W , Kim G . A functional bioink and its application in myoblast alignment and differentiation. Chem Eng J. 2019;366:150‐162.

[45] Sharma RI , Snedeker JG . Paracrine interactions between mesenchymal stem cells affect substrate driven differentiation toward tendon and bone phenotypes. PloS One. 2012;7:e31504. 22355373 10.1371/journal.pone.0031504 PMC3280320

[46] Conti FJ , Monkley SJ , Wood MR , Critchley DR , Müller U . Talin 1 and 2 are required for myoblast fusion, sarcomere assembly and the maintenance of myotendinous junctions. Development. 2009;136:3597‐3606. 19793892 10.1242/dev.035857 PMC2761109

