# Supplementary material for: A bioprinted complex tissue model for myotendinous junction with biochemical and biophysical cues
Source: Bioeng Transl Med. 2022 Apr 5;7(3):e10321. doi: 10.1002/btm2.10321 (PMC9472009; doi:10.1002/btm2.10321)
Supplement: Supplementary file 1 — Appendix S1 Supporting Information [file BTM2-7-e10321-s001.docx]

**SUPPLEMENTAL METHODS**

**Decellularization of porcine muscle and tendon tissue**

The isolated tissues were cut into small cubes (smaller than 8 × 8 × 3 mm^3^) and rinsed three times in Dulbecco’s phosphate-buffered saline (DPBS; Biowest, USA). These tissues were decellularized by treatment with 1% (w/v in DPBS) sodium dodecyl sulfate (SDS; Sigma-Aldrich) solution for 5 days, 1% (in DPBS) Triton X-100 (Sigma-Aldrich) for 2 days, 1% (in DPBS) PS for 1 h, DNase I solution (Sigma-Aldrich) for 2 h, rinsed three times with DPBS and deionized water, and freeze-dried using a lyophilizer (SFDSM06; Samwon, South Korea). The lyophilized skeletal muscle dECM (mdECM) and tendon dECM (tdECM) were stored at −80 °C before solubilization.

To solubilize the mdECM and tdECM, the dried specimens (10 mg dECM per 1 mL pepsin solution) were digested in 0.1 % (w/v in 0.5 M acetic acid; Sigma-Aldrich) pepsin solution (Sigma-Aldrich) at room temperature for 2 days. Following the precipitation of the digested tissues by the addition of sodium chloride (Sigma-Aldrich), dialysis was performed using a 1000-kDa molecular cut-off (MCO) tube (Spectrum Chemical Manufacturing, USA) at 4 °C for 3 days. The dialyzed soluble mdECM and tdECM were lyophilized and stored at −80 °C. To estimate the DNA and ECM contents of the mdECM and tdECM, PicoGreen (Thermo-Fisher Scientific), soluble collagen (Biocolor Live Sciences Assays, UK), Blycan sulfate glycosaminoglycans (GAGs) (Biocolor Live Sciences Assays, UK), and Fastin elastin (Biocolor Live Sciences Assays, UK) assay kits were used according to the manufacturer’s protocols and previously described protocols.

**Degradation analysis**

The degradation rate of the M-bioink, T-bioink, collagen, and MTJ structures was assessed using collagenase solution. Following measurement of the initial weight (M_0_) of the lyophilized specimens, those were incubated in the collagenase solution (0.1 in DPBS; Sigma-Aldrich) for 0.5, 1, 3, 5, 7, and 14 d. At each time point, the incubated samples were rinsed with deionized water, lyophilized, and weighed (M_t_). Following equation was used to calculate the mass loss:

Mass loss = [(M_0_ – M_t_)/M_0_] × 100

The values of estimated mass loss are shown as the mean ± SD (n = 4).

**SUPPLEMENTAL FIGURES**


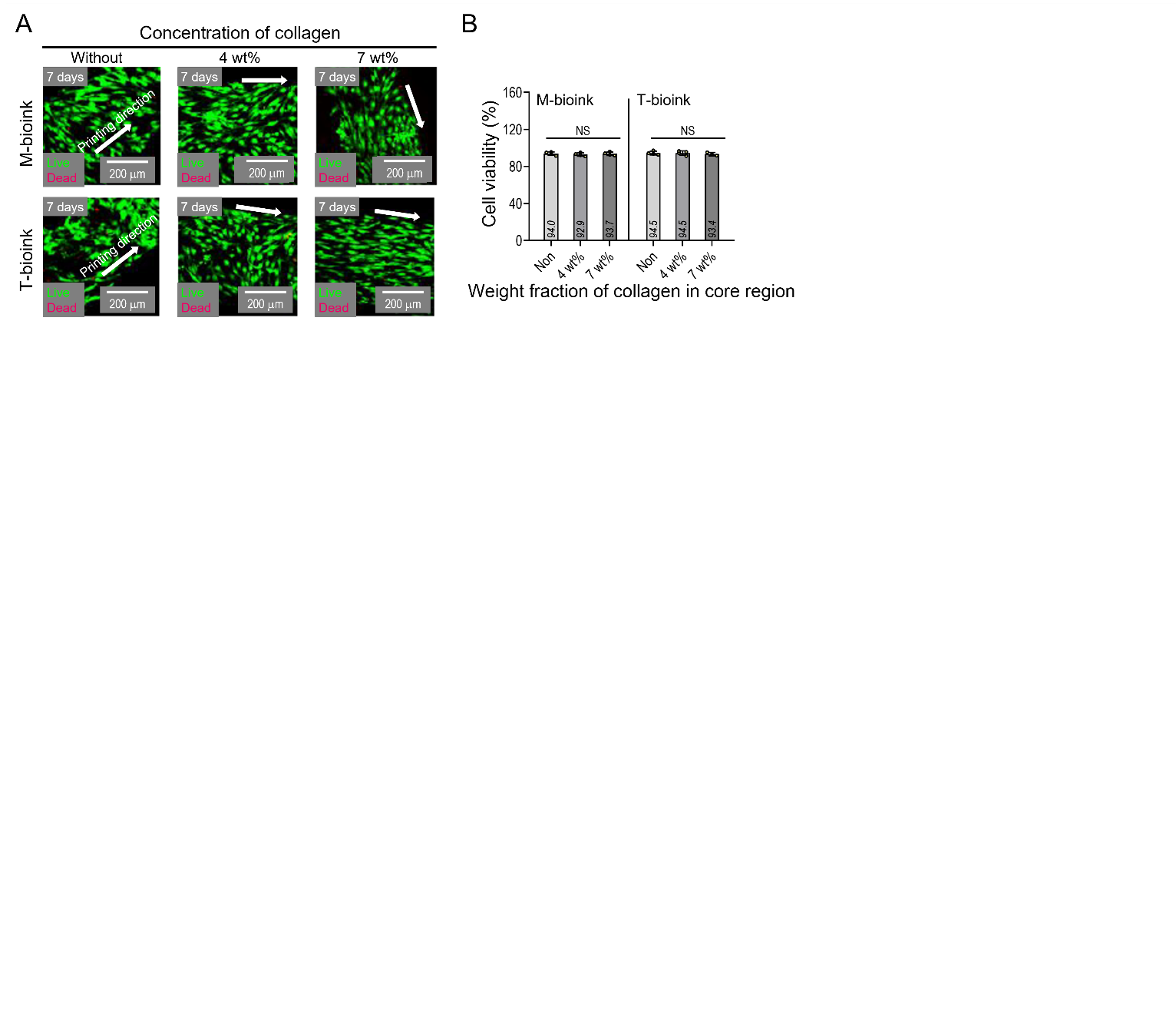


Figure S1. Effects of concentration of collagen (core channel) on cell viability observed in (A) live (green)/dead (red) images and (B) cell viability calculated using the images. (n < 4)


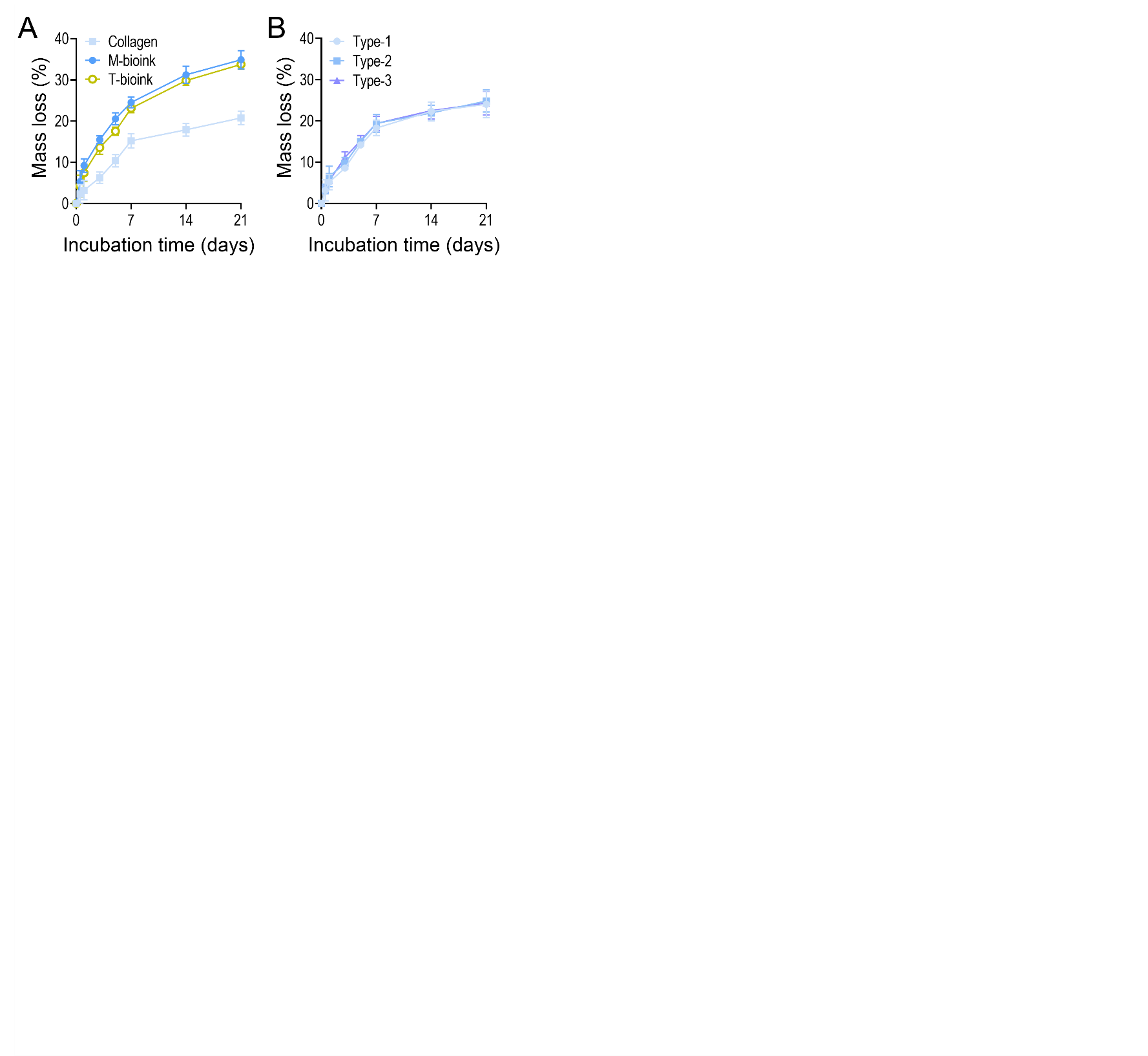


Figure S2. Biodegradation of (A) the constructs fabricated using cell-free collagen and M- and T-bioinks and (B) MTJ constructs in the collagenase solution (0.1 U/mL). (n = 4)
